# Anti-protein immunoglobulin M responses to pneumococcus are not associated with aging

**DOI:** 10.1186/s41479-018-0048-3

**Published:** 2018-06-05

**Authors:** Esther L. German, Bahij Al-Hakim, Elena Mitsi, Shaun H. Pennington, Jenna F. Gritzfeld, Angie D. Hyder-Wright, Antonia Banyard, Stephen B. Gordon, Andrea M. Collins, Daniela M. Ferreira

**Affiliations:** 1 Respiratory Infection Group, Liverpool School of Tropical Medicine, Liverpool, UK; 2 Royal Liverpool University Hospital, Liverpool, UK; 3 Present address: Aintree University Hospital, Liverpool, UK; 4 Present address: Public Health England, Vaccine Evaluation Unit, Manchester, UK; 5 Present address: Cancer Research UK Manchester Institute, Manchester, UK; 6 Present address: Malawi-Liverpool-Wellcome Trust, Blantyre, Malawi

**Keywords:** IgM antibodies, Pneumococcus, Vaccines, Immune senescence, Proteins

## Abstract

**Background:**

The incidence of community-acquired pneumonia and lower respiratory tract infection rises considerably in later life. Immunoglobulin M (IgM) antibody levels to pneumococcal capsular polysaccharide are known to decrease with age; however, whether levels of IgM antibody to pneumococcal proteins are subject to the same decline has not yet been investigated.

**Methods:**

This study measured serum levels and binding capacity of IgM antibody specific to the pneumococcal surface protein A (PspA) and an unencapsulated pneumococcal strain in serum isolated from hospital patients aged < 60 and ≥ 60, with and without lower respiratory tract infection. A group of young healthy volunteers was used as a comparator to represent adults at very low risk of pneumococcal pneumonia. IgM serum antibody levels were measured by enzyme-linked immunosorbent assay (ELISA) and flow cytometry was performed to assess IgM binding capacity. Linear regression and one-way analysis of variance (ANOVA) tests were used to analyse the results.

**Results:**

Levels and binding capacity of IgM antibody to PspA and the unencapsulated pneumococcal strain were unchanged with age.

**Conclusions:**

These findings suggest that protein-based pneumococcal vaccines may provide protective immunity in the elderly.

**Trial registration:**

The LRTI trial (LRTI and control groups) was approved by the National Health Service Research Ethics Committee in October 2013 (12/NW/0713). Recruitment opened in January 2013 and was completed in July 2013. Healthy volunteer samples were taken from the EHPC dose-ranging and reproducibility trial, approved by the same Research Ethics Committee in October 2011 (11/NW/0592). Recruitment for this study ran from October 2011 until December 2012. LRTI trial: (NCT01861184), EHPC dose-ranging and reproducibility trial: (ISRCTN85403723).

## Background

Community-acquired pneumonia (CAP) has an incidence of 5–11 per 1000 adult population [1, 2], with an associated mortality between 5 and 20%, increasing to 50% in cases admitted to the intensive care unit [3]. The most common cause of CAP is *Streptococcus pneumoniae* [4]. Eighty per cent of CAP occurs in people ≥60 years [5–7], and this has been attributed to immune senescence, impairment of protective airway reflexes [8], reduced airway mucociliary clearance [9, 10] and alterations in respiratory tract immune function [11, 12]. Polysaccharide-based vaccines are recommended for older adults: the pneumococcal conjugate vaccine (PCV) is now licensed for adults over the age of 50 in the United States (US) and the pneumococcal polysaccharide vaccine (PPV) is recommended to people over 65 years of age in the United Kingdom (UK). However, the true levels of protection conferred by these vaccines in older adults are unclear [13–16]. They also have significant short-comings with regards to limited pneumococcal serotype coverage. Protein-based vaccines are being investigated as alternative immunization strategies, on the basis that they could provide full serotype coverage by targeting conserved proteins. As immunogenicity in vivo will depend on exposure to the host immune system, surface-expressed proteins are obvious potential vaccine candidates.

The protective role of antibody responses to pneumococcal infection is well described. IgM antibodies are the first class of antibodies to be produced in a primary response to microbial challenge. Although they bind with low affinity they exhibit a broad specificity to a variety of antigens [17]. Murine models have demonstrated that the passive transfer of IgM antibodies targeting pneumococcal polysaccharides can improve survival following a lethal pneumococcal challenge, suggesting that IgM antibody may be sufficient to confer protection against pneumococcal disease [18]*.* The therapeutic potential of an immunoglobulin preparation enriched with IgM for the treatment of severe CAP is under investigation [19]. Levels of antibodies to pneumococcal polysaccharides decline with aging, with IgM levels being more affected than the IgG subclass [20–22]. Levels of IgG to several pneumococcal protein antigens are also reduced in the elderly [22].

Pneumococcal surface protein A (PspA) is a major and well characterized virulence factor of the pneumococcus. Antibodies directed against PspA augment C3 deposition, and immunization with PspA confers protection to a variety of murine infection models [23, 24]. PspA is expressed in the majority of clinically important pneumococcal serotypes [25] and clade 4 in particular exhibits broad cross reactivity [26], allowing for sensitive detection of anti-PspA antibodies.

Despite evidence that IgM antibodies may play a role in anti-pneumococcal defence, and that IgG antibodies to pneumococcal proteins have protective potential, the impact of age on levels of IgM antibodies to pneumococcal proteins in humans has not been investigated. This study seeks to address this knowledge gap by investigating changes in levels and function of IgM antibodies against pneumococcal proteins in relation to aging and lower respiratory tract infection (LRTI) using the PspA clade 4 (PspA4) and an unencapsulated pneumococcal strain. It was hypothesised that as subjects age, levels and binding capacity of IgM antibodies against pneumococcal proteins in serum would be impaired and that patients hospitalized with LRTI would have lower IgM levels and functionality when compared to controls.

## Methods

### Study subjects

Serum samples used in this study were collected as part of two clinical trials, both of which have already had their primary end-points reported elsewhere: the LRTI trial [27] and the dose-ranging trial [28]. The volunteers can be separated into three cohorts: LRTI, control (both from the LRTI trial) and healthy (taken from the dose-ranging trial).

The LRTI cohort consisted of patients with a diagnosis of LRTI, based on presentation with symptoms of respiratory infection with ≥2 of the following clinical signs: “cough, breathlessness, pleuritic chest pain, fever and increased or new sputum production” [27]. Without additional radiological consolidation, LRTI patients cannot be considered to have CAP, but this broader cohort was chosen because of its clinical relevance in the UK hospitals setting [27].

The control cohort comprised patients admitted to hospital for reasons other than LRTI. They had no respiratory symptoms, and were age- and gender-matched to the LRTI patients.

The healthy cohort was formed of healthy volunteers aged 18–60 with no concurrent co-morbidities.

### Sample collection

Nasal washes and serum samples were taken from LRTI and control group volunteers within 3 days of hospital admission. Baseline nasal washes and serum samples were taken from healthy volunteers by appointment.

Nasal washes were processed as described previously [27, 28] and used to determine pneumococcal carriage status and density by classical microbiology and by *lytA* qPCR. Urine from LRTI and control volunteers was collected to perform the BinaxNOW (Alere International Ltd., Ireland) (urine chromatography) test.

### Bacterial strains

An unencapsulated D39 strain with deleted *cpsD* (D39-D∆) [29] (donated by Professor Jeremy S. Brown, University College, London) was cultured in Todd-Hewitt broth with 0.5% yeast extract (THY) until the mid-log phase. Bacteria was then pelleted, washed in phosphate buffered saline (PBS) and either used for whole cell ELISA (WCE) or resuspended in THY+ 20% glycerol and stored at − 80 °C for later use in an immunoglobulin binding assay.

### Enzyme-linked immunosorbent assay (ELISA)

ELISA was used to quantify levels of IgM antibodies in the serum samples as previously described [30]. Briefly, 96-well plates (Nunc, Denmark) were coated with carbonate/bicarbonate buffer containing capture antigens/antibodies. The capture antigens/antibodies included 1 μg/ml recombinant PspA4 (kindly donated by Dr. Eliane Miyaji, Butantan Institute, Sao Paulo, Brazil), the non-encapsulated D39-D∆ pneumococcal strain and anti-human IgM μ chain-specific antibody (I2386, Sigma-Aldrich, St Louis, Missouri, USA).

Plates were washed then blocked with 1% bovine serum albumin(BSA)-PBS before adding serial dilutions of serum samples. Standard curves for calculating total IgM were made using purified total IgM from serum (I8260, Sigma-Aldrich, USA) at a level of 1 mg/ml. WCE and anti-PspA4 values were based on a standard pool serum with an IgM level of 4000 arbitrary units/μl (donated by Prof David Briles, University of Alabama).

IgM was bound to a goat anti-human IgM polyclonal antibody conjugated to biotin (Abcam, Cambridge, UK). The conjugate was quantified by streptavidin-alkaline phosphatase (Bio-Rad Laboratories, Hemel Hempstead, UK) using 4-Nitrophenyl phosphate disodium salt hexahydrate (N9389, Sigma-Aldrich, USA) as the substrate for development. Absorbance at 405 nm wavelength was determined using the Fluostar Omega® (BMG Labtech, Ortenberg, Germany) plate reader. A standard curve was fitted using a 4-parameter fit model.

### Flow cytometry

Immunoglobulin deposition on whole live unencapsulated pneumococci was assayed using a modification of a published flow cytometry assay [31]. Briefly, D39-DΔ resuspended in THY + 20% glycerol was grown to mid-log phase in THY media with erythromycin, before two cycles of washing with PBS. Serum from patient groups was pooled and diluted in PBS at ratios of 1:2 and 1:20. Fixed volumes of the bacterial suspension were washed and resuspended in 100 μl of pooled serum before incubation for 30 min at 37 °C. Samples were pelleted, resuspended in IgM-PE-Cy5 (Clone G20–127, BD Biosciences, Franklin Lake, NJ, USA) and incubated for 20 min at 37 °C. Bacteria were washed and the level of IgM binding measured by flow cytometry using a BD LSR II (BD Biosciences, New Jersey, USA). The level of binding was calculated as the percentage of the bacterial population positive for IgM deposition multiplied by the geometric mean fluorescence of the bacterial population positive for IgM.

### Statistical analysis

Linear regression analysis was used to correlate levels of antibodies with age. Antibody levels were log transformed to obtain normal distribution then analysed by one-way ANOVA with the Bonferroni correction to compare levels between the three groups. Analyses were performed using GraphPad Prism v5 (GraphPad Inc., California, USA) and significance was set at *p* < 0.05.

## Results

### Clinical trials

Overall, 38 hospital inpatients were enrolled in the LRTI trial, equally divided between patients with a diagnosis of LRTI (*n* = 19) and controls (*n* = 19). All LRTI patients, and 3 control patients, had been treated with antibiotics before recruitment to this study [27]. However, none of the control patients presented with a symptomatic infection. 10 volunteers were chosen from the dose-ranging study to be included in this analysis as healthy volunteers.

Table 1 outlines cohort characteristics. The mean age for LRTI patients was 64.5 years ±15.8 and 64.6 years ±14.5 for the control group. The mean age of the healthy group was 21.2 years ±2.4. A similar percentage of volunteers were female in all three cohorts: 53% for LRTI and control groups, and 50% for healthy volunteers. Risk factors for pneumococcal infection were recorded for both LRTI and control cohorts. Vaccination with PPV and contact with children was similar between both groups, whereas LRTI patients were more likely to be smokers, use corticosteroids and have COPD and/or asthma (Table 1).

**Table 1:** Study population characteristics

	LRTI (*n* = 19)	Controls (*n* = 19)	Healthy volunteers (*n* = 10)
Age (mean ± SD) (yr)	64.5 (15.8) 10 (53)	64.6 (14.5) 10 (53)	21.2 (2.4) 5 (50)
Female gender (n [%])			
Smoker/ex-smoker (n [%])	15 (79)	10 (53)	0 (0)
PPV (n [%])	7 (37)	8 (42)	0 (0)
Contact with children (n [%])	10 (53)	12 (63)	0 (0)
Corticosteroid use (n [%])	16 (84)	3 (16)	0 (0)
COPD (n [%])	8 (42)	2 (11)	0 (0)
Asthmatic (n [%])	5 (26)	1 (5)	0 (0)

COPD, chronic obstructive pulmonary disease; LRTI, lower respiratory tract infection; PPV, pneumococcal polysaccharide vaccine

Evidence was found of recent pneumococcal exposure in 50% (19/38) of the study’s patients, either by detection of pneumococcal nasal carriage or BinaxNOW (urine immunochromatography) test [27]. Of these, 11 belonged to the LRTI cohort. There was no significant difference in IgM antibody levels between pneumococcal carriers and non-carriers, nor was there any correlation between IgM antibody levels and carriage density by qPCR (data not shown).

### Effect of age on immunoglobulin M antibody levels in serum

**Fig. 1:**
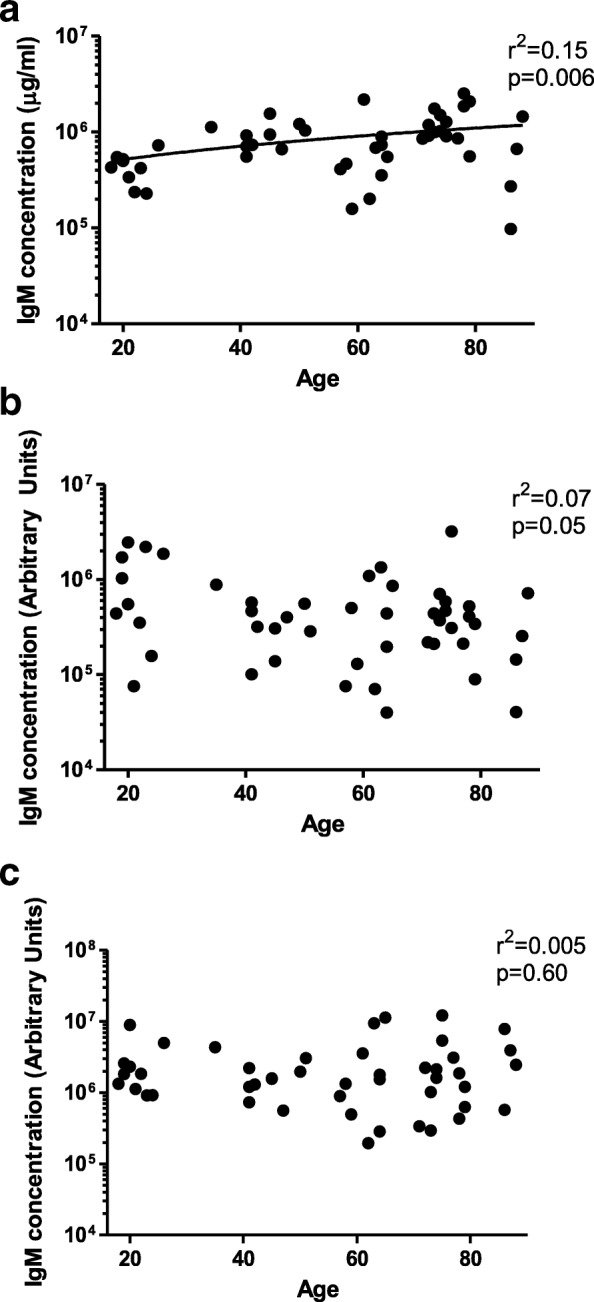
Levels of IgM in relation to age. Levels of (**a**) total IgM, (**b**) IgM to the unencapsulated D39-ΔD pneumococcus and to the (**c**) Pneumococcal Surface Protein A (PspA4) were plotted against volunteer age. Correlations were analysed using linear regression analysis

While the total levels of IgM trended to increase with increase of age (r^2^ = 0.15, *p* = 0.006) (Fig. 1a), no correlation was observed for levels of specific pneumococcal IgM antibody and age: D39-DΔ (r^2^ = 0.07, *p* = 0.05) and PspA4 (r^2^ = 0.005, *p* = 0.60) (Figs. 1b and c).

### Effect of lower respiratory tract infection on immunoglobulin M levels

**Fig. 2:**
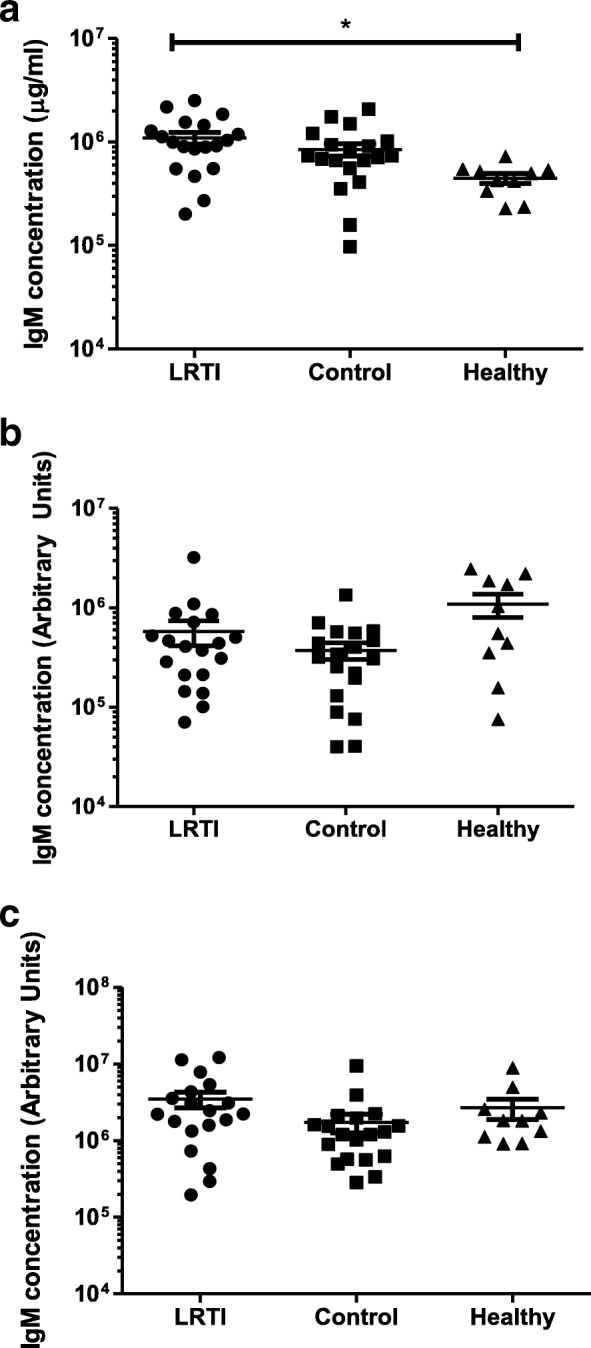
Levels of IgM in relation to LRTI. The level of total IgM was examined for patients with LRTI, hospital Controls and a young cohort of Healthy volunteers. Levels of (**a**) total IgM, (**b**) IgM to the unencapsulated D39-ΔD pneumococcus and to the (**c**) Pneumococcal Surface Protein A (PspA4) are shown. Mean values ± SD are shown. * *p* < 0.05 using ANOVA with Bonferroni’s correction

There was no difference in total IgM levels between LRTI and control groups. The LRTI group had higher levels of total IgM when compared to the healthy group (*p* = 0.01 for ANOVA analysis, *p* < 0.05 for the difference between LRTI and healthy groups) (Fig. 2a). No significant difference was observed in IgM antibody levels to D39-DΔ (*p* = 0.64 for ANOVA analysis) or to PspA4 (*p* = 0.14 for ANOVA analysis) between any of the groups (Figs. 2b and c).

### Immunoglobulin binding to whole-cell unencapsulated pneumococcus

**Fig. 3:**
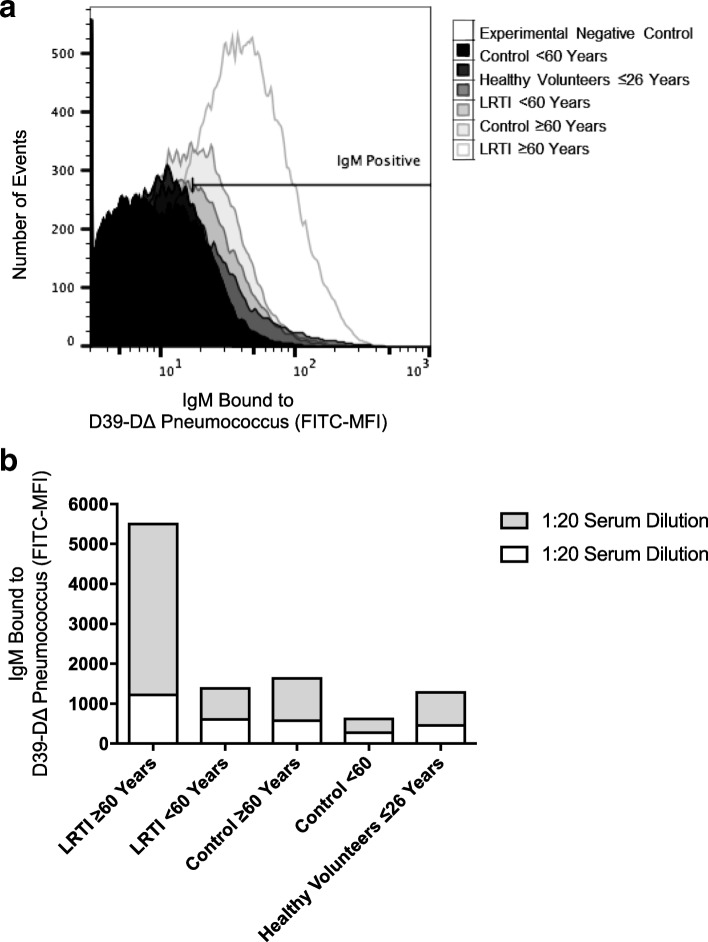
Flow cytometric assessment of IgM binding to pneumococcus surface. Pooled sera from different volunteer groups were added to bacteria. Two experiments using different dilutions (1:2 and 1:20) of sera were performed. **a** Histogram of surface binding of IgM to intact pneumococcus D39-D∆. **b** Surface binding of IgM from pooled serum samples expressed as the percentage of the bacterial population positive for IgM deposition multiplied by the geometric mean fluorescence of the bacterial population positive for IgM

The immunoglobulin binding assay was performed to assess changes in the binding capacity of IgM with aging and LRTI status. Sera from ≥60 control group showed no decrease in binding compared to younger volunteers from the healthy group (Figs. 3a and b). Contrary to the study’s initial hypothesis, the ≥60 LRTI group exhibited the highest level of IgM binding while the < 60 control group showed the lowest levels of binding.

## Discussion

These results demonstrate that the levels and binding capacity of IgM to pneumococcal proteins are unaltered with aging. Patients with LRTI did not show decreased IgM antibody to D39-DΔ or PspA4 compared to either the control or healthy groups. The study observed higher IgM binding to pneumococcus in a flow cytometry assay for sera from LRTI patients over 60 years.

High levels of total IgM in patients with LRTI cannot be an artefact of current infection, but rather of aging, because control patients without infection presented with similar total IgM levels, but the healthy group had significantly lower total IgM levels. Although only IgM antibodies against pneumococcal polysaccharides and proteins can be expected to confer protection against pneumococcal infection, it is nevertheless interesting to note that total IgM antibody levels are maintained despite immune senescence.

A study of IgM levels in mice found that total IgM levels were increased in older animals [32]. However, the authors found that anti-pneumococcal protein IgM antibody levels (as indicated by antibodies to phosphoryl choline, a pneumococcal cell wall antigen) dropped with age. This discrepancy with the present study’s data could be explained by the different choice of antigen or the limitation of animal models (which are immunologically naïve) to represent human responses (which are frequently re-challenged by in vivo exposure to pneumococcus through nasal carriage) [28]. IgM from older mice (18- or 24-month) was less protective against pneumococcal challenge when compared to younger mice (3-month) [32]. The present study showed, however, that IgM in adults over the age of 60 retains the ability to bind to non-encapsulated *S. pneumoniae*.

In whole-cell ELISA, in addition to surface protein antigens, cell wall antigens are also present, to which IgM binding may occur; however, whether these antibodies confer meaningful protection has been questioned [33, 34]. It has been suggested that bacterial autolysis during the overnight coating step releases internal antigens which would not in vivo be presented to the immune system [31]. Antibodies to these internal and cell wall antigens could conceivably lead to an overestimation of levels of IgM antibody against surface protein antigens. To address this issue, the study also used a flow cytometric approach to evaluate antibody binding to intact pneumococcus. Whereas cohort and age showed no impact on antibody levels measured by whole cell ELISA, differences were observed in antibody binding as assessed by flow cytometry. This discrepancy could be explained by bacterial rupture.

Limitations of this study include the small number of samples and the unknown etiology of LRTI of these patients. This study cohort also had significant heterogeneity with respect to many factors affecting immunoglobulin levels; for example, vaccination status, co-morbidities, corticosteroid treatment, and history of previous pneumococcal infection [27]. Furthermore, 12 of the LRTI patients received Clarithromycin prior to recruitment. The activities of 14-membered macrolide antibiotics are known to exert immunomodulatory effects [35] and this may have affected IgM responses to *S.pneumoniae* in this cohort.

## Conclusions

In summary, these findings suggest that IgM antibodies against pneumococcal proteins do not decrease with age and this warrants further investigation into IgM antibodies against pneumococcal proteins and its role in immunity through pneumococcal protein vaccination.

## Abbreviations

CAP: Community Acquired Pneumonia
D39-D∆: Unencapsulated D39 strain with deleted *cpsD*
LRTI: Lower Respiratory Tract Infection
PBS: Phosphate Buffered Saline
PCV: Pneumococcal Conjugate Vaccine
PPV: Pneumococcal Polysaccharide Vaccine
PspA: Pneumococcal surface protein A
THY: Todd-Hewitt broth with 0.5% yeast extract
WCE: Whole Cell ELISA
